# Developing medical oncology in Africa

**DOI:** 10.3332/ecancer.2019.940

**Published:** 2019-07-25

**Authors:** Naftali Busakhala

**Affiliations:** Moi University School of Medicine, PO Box 4606-30100, Eldoret, Kenya

**Keywords:** medical oncology, integration, collaboration

## Abstract

**Background:**

Kenya, like most other developing countries, is undergoing an epidemiologic shift of disease patterns characterized by an increasing prevalence of cancer and other non-communicable diseases straining health care resources which were mainly intended for communicable diseases. We describe the development of sustainable cancer prevention and control programs at Moi University and Moi Teaching and Referral Hospital in Western Kenya.

**Methods:**

The cancer prevention and control program at Moi Teaching and Referral Hospital was started by volunteer nurses and clinicians in 2005 in response to a high prevalence of AIDS-related Kaposi sarcoma. Chemotherapy was donated by a local drug store until 2007 when Eli Lilly pharmaceuticals from Indianapolis in the USA started helping the program through the Academic Model Providing Access to Healthcare (AMPATH). Due to good response rates of patients with AIDS-related Kaposi sarcoma, and lobbying by volunteers, the service became a formal department of Moi Teaching and Referral hospital in 2008.

**Results:**

The department has now grown to become the second largest public cancer centre in the country registering about 9000 patient visits per year. In addition, staff have now specialized in various areas such as medical oncology, palliative care, surgical oncology, nursing and gynaecology oncology.

**Conclusions:**

The development of a medical oncology program requires a multi-disciplinary team focused on integration within existing programs and expansion of collaborative networks in order to provide the best care to patients.

## Introduction

Cancer is the second most common cause of mortality after cardiovascular diseases globally [1]. It is estimated that 18.1 million people will develop cancer and 9.6 million people will die from cancer in 2018 [2]. Men are more likely to develop cancer between birth and age 79 years, with one in five men and one in six women developing cancer worldwide and one in eight men and one in ten women dying from it [2].

Africa accounts for 5.8% of the global incidence of cancer but 7.3% of the mortality, reflecting a higher death rate compared to Europe and America [2]. It is projected that in 2030, the population of Africa will be about 1.56 billion people with a corresponding increase in the incidence of cancer to 1.27 million cases and a mortality rate of 0.97 million deaths [3] Africa is, therefore, facing a cancer epidemic which requires proper planning and resource allocation to develop cancer-control programmes. The response to the cancer epidemic in sub-Saharan Africa has been affected by underfunding and inefficiency in health systems. In April 2001, African heads of state met in Abuja, Nigeria and resolved to allocate at least 15% of their annual revenues to health as a part of the Millennium Development Goals. After 10 years, only one country had attained the target. Three other countries were on track, while the rest have no or insufficient progress [4]. Kenya allocates less than 6% of the national revenue to health, leading to inadequate resources for the entire health system including oncology. In addition to national revenues, the Abuja declaration requires increased collaboration with donors and improved efficiency of resources. In this paper, we describe the ongoing collaborative development of a cancer control and prevention programme at Moi Teaching and Referral Hospital (MTRH)/Moi University, cascaded from the National Cancer Control Strategy 2017–2022 and implemented within the MTRH/Moi University framework.

## Mortality trends in Kenya

**Figure 1.**
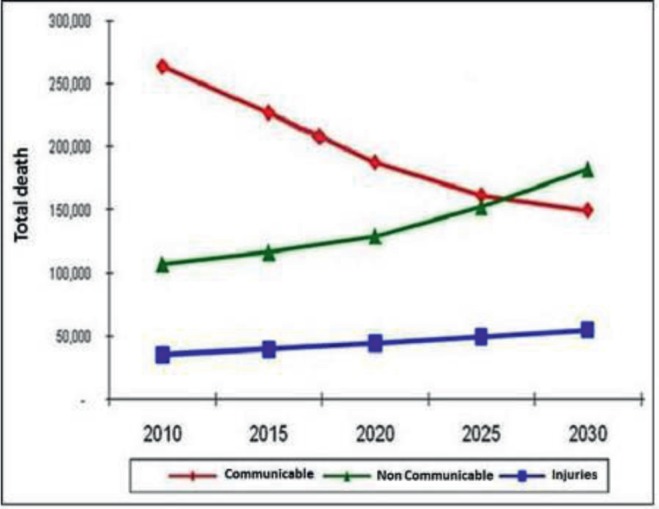
Mortality trends in Kenya. Source: Ministries of Medical Service, Public Health and Sanitation, Comprehensive National Health Policy Framework, 2011 [7] (unpublished data).

Kenya, like most other developing countries, is undergoing an epidemiologic shift of disease patterns characterised by increasing prevalence of cancer and other non-communicable diseases and a double strain on health care resources. These emerging lifestyle and genetic diseases previously associated with high-income countries are not replacing infectious diseases but adding to them [5, 6] (Figure 1). Cancer is ranked as the third most common cause of death in Kenya, after infectious and cardiovascular diseases. The annual incidence is about 40,000 cases with mortality estimated at 28,000 cases (Ministry of Health, 2018).

## A systems approach

Prior to 2005, there was no formal oncology service at MTRH. We decided to prevent and control cancer by using infrastructure already built for prevention and control of infectious diseases. This is particularly important because most of the common cancers, such as cervical cancer, Kaposi’s sarcoma, lymphomas, liver and stomach cancer are caused by infections and can be prevented [8]. The most common cancer in MTRH at that time was AIDS-related Kaposi’s sarcoma, and therefore we worked with the Academic Model Providing Access to Health Care (AMPATH) which was providing HIV services. AMPATH is a collaborative effort between Moi University, MTRH and a consortium of North American Universities led by Indiana University [9]. Oncology services integrated easily into the system because they were complementary to AMPATH.

### Building consensus among stake holders

We called for meetings with everyone who was interested in cancer care, including prevention, diagnosis, education, treatment and palliative care. Prior training was helpful, but not necessary because we planned to train people through apprenticeship and formal programmes once the service had officially started. All the stakeholders from MTRH, Moi University, Ministry of Health and collaborating partners were involved. The main objective was building consensus, networks and inspiring other specialties to grow.

## Medical oncology

Medical oncology is a speciality that deals with diagnosis and treatment of adults with cancer using chemotherapy, hormonal therapy, biological therapy and targeted agents. The other specialities in oncology include surgical oncology, radiation oncology, paediatric oncology, nuclear medicine and others. A medical oncologist coordinates diagnostic work-ups, laboratory analyses, referrals to sub-specialities, pain management, hydration, nutrition and best supportive care. Almost every adult cancer patient will require medical oncology. We recognised that medical oncology cannot develop in isolation. It requires pathologists, surgeons, nurses, gynaecologists, nephrologists, pulmonologists, dermatologists and others. The strategy was to work in harmony with all sections of the hospital in order to garner goodwill.

### Integration into hospital administrative structure

**Figure 2.**
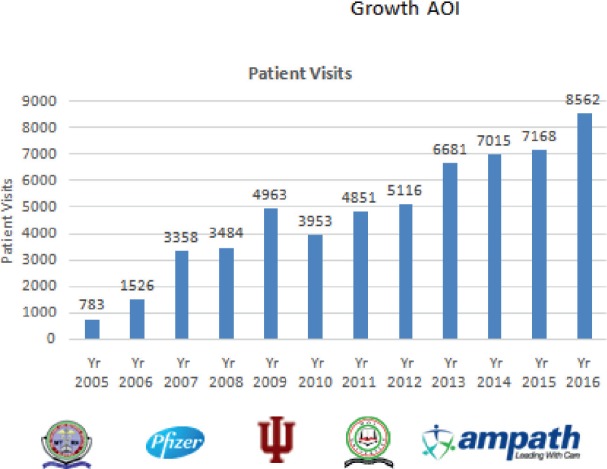
Growth of haematology-oncology services.

This involved formal integration into the hospital structure and representation at all levels of hospital management. Members of staff got appointment letters with a clear mandate to prevent and control cancer both in the hospital and in the catchment area. The hospital provided clinic space, diagnostic and therapeutic supplies. The hospital also recruited personnel and allowed interested staff to take paid study leaves to train as haematologists and oncologists in line with the staff development policy (Figure 2). Finally, the hospital created a Department of Oncology which has grown over the years with corresponding increasing budgetary allocation.

## Staff development

**Figure 3.**
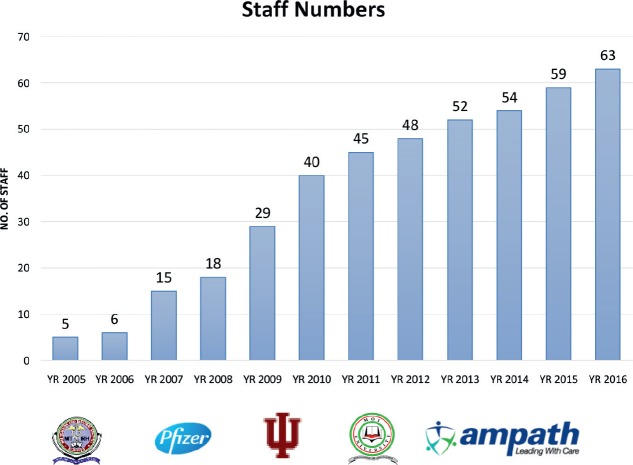
Growth of human resources.

Staff acquired oncology knowledge and skills through working with preceptors from North America, mainly Indiana and Brown University. Exchange programmes allowing local staff to go for observership in North America expanded their knowledge, while working together locally enabled them to gain practical skills. Among the early beneficiaries of this process are three gynaecologists who eventually drafted a curriculum for Master of Science in Gynaecology-Oncology. This curriculum was approved by Moi University Senate with recognition from all professional bodies in the country. The success of this programme has led to development of a post-graduate curriculum in medical, paediatric, nursing and radiation oncology at Moi University. Non-graduate nurses and clinical officers holding diploma qualifications are able to advance through the hospital training school, where we have developed a higher national diploma in nursing and medical oncology. Through this process, we have trained at least 20 core staff members, comprising medical oncologists, oncology nurses, oncology pharmacists and pharmacy technologists, palliative care specialists and cancer registrars (Figure 3).

The long-term objective is to be a centre of excellence both in the training of personnel and actual prevention and control of cancer [10].

## Research and publications

Both retrospective and prospective observational and interventional studies are routinely conducted in the Directorate of Haematology and Oncology. The majority of the studies are local investigator-initiated. We are actively involved in multi-centre trials as part of the AIDS-Clinical Trial Group and AIDS Malignancy Consortium. Participation in these trials has helped to improve the standards of care as well as providing funds for purchase of equipment.

## Goals of the oncology programme

1. Provide high-quality integrated and multidisciplinary care, including palliation for patients with blood disorders and cancer
2. Integrate clinical care with teaching and research in blood disorders and cancer
3. Promote cancer prevention and early detection, surveillance and improve diagnosis
4. Promote partnerships and collaboration between MTRH, Moi University, Indiana University and other institutions
5. Promote the centre’s services to the public and integrate cancer-control activities in primary healthcare

## Strategic planning

We have an active cancer prevention and early detection programme both at the hospital and in the community. We have four main outreach sites in rural community hospitals where clients are continuously educated on cancer prevention and screened for early detection of cancer. The efforts involving cancer prevention and early detection focus on breast, uterine cervix, liver and skin cancers but also address other diseases depending on the questions asked by clients.

Management is based on current ESMO and/or NCCN guidelines. Treatment starts with counselling of the patient followed by disclosure to family if the patient consents. Alterations to standard protocols are discussed and agreed during tumour board meetings. Use of protocols makes treatment predictable for patients, clinicians and pharmacists. This allows purchase of drugs and supplies in bulk, hence lowering the overall cost of care.

We have developed and implemented innovations, such as task-sharing, where general nurses and clinical officers are trained to perform specialised oncology duties, and out-patient blood transfusion services. We have developed a training curriculum for oncology nurses offered at the MTRH training school. The curriculum has been approved by the Ministry of Health which confers national recognition to the trainees. We have also developed an oncology training curriculum for Clinical Officers. The Clinical Officers are Diploma holders with basic skills who work under the supervision of medical doctors in health care delivery. Upon completion of training, both nurses and clinical officers are offered a Diploma in Oncology and are employed by both public and private institutions. This has provided a stop-gap measure as the country still trains specialised oncology doctors.

## Challenges

Lack of local research funding has deprived the programme of an opportunity to understand the characteristics of local oncology patients. These include genetic variations in cancer, ability to ascertain diagnostic accuracy, monitoring effectiveness of treatment and survival. Most research is externally funded focusing on objectives that may not be a priority locally or lacking post research care and follow-up. The government, through the National Commission for Science and Innovation, allocates limited funding for research and hopefully this will be increased in future.

High rates of loss-to-follow-up of patients have made it difficult to implement treatment protocols effectively. Medications that should be administered every 3 weeks can sometimes be administered every 6 weeks because that is when patients show up. Sometimes patients get lost completely preferring to get medication from herbalists and faith healers. We are engaging community health workers to solve this problem. We are also encouraging our patients to enrol in the National Hospital Insurance Fund because insurance will reduce the number of patients lost to follow-up due to lack of funds.

Lack of radiation services at MTRH causes most patients to be referred to Kenyatta National Hospital (KNH) in the capital city Nairobi which is 320 km away. That is the only hospital where public radiotherapy equipment is available. Once they arrive at KNH, they are given long booking periods because service is provided on a ‘first-come–first-served’ basis and the demand is higher than the radiation equipment can handle. Most of these patients rarely get radiotherapy. To solve this problem, Indiana University in the USA has assisted MTRH to construct two bunkers for radiation equipment. The hospital is currently working with the International Atomic Energy Agency to buy a linear accelerator and brachytherapy equipment. Lack of training facilities has limited the number of students who can be trained. This is being solved by hiring the nurses and clinical officers upon completion of oncology training. There are also plans to increase classrooms and expand oncology wards to expand training capacity.

## Conclusions and lessons learned

The success of a medical oncology service depends on how well it integrates with other existing hospital services, including administrative services. Medical oncology and other specialities develop in synergy with health care services. At MTRH, we developed the programme using AMPATH infrastructure initially built for HIV care.

Collaborative networks are important to the growth of oncology services in Africa. As earlier explained, most African countries have insufficient resources to develop oncology programmes and need donor support to initiate and sustain these services despite commitments made in the Abuja declaration. The cost of diagnosing and treating cancer is beyond the financial means of most patients in Africa, including those covered by medical insurance.

## Abbreviations

AMPATH: Academic Model Providing Access to Healthcare; MDGs: Millennium Development Goals; MTRH: Moi Teaching and Referral Hospital; HIV: Human Immune Deficiency Virus; AOI: Ampath Oncology Institute; ACTG: AIDS Clinical Trials Group; AMC: AIDS Malignancy Consortium; ESMO: European Society of Clinical Oncology; NCCN: National Comprehensive Cancer Network; NHIF: National Health Insurance Fund; KNH: Kenyatta National Hospital

## Funding

The author did not receive any funding for this article.

## Conflicts of interest

The author has no conflicts of interest to report.
